# Egr-1 Regulates Autophagy in Cigarette Smoke-Induced Chronic Obstructive Pulmonary Disease

**DOI:** 10.1371/journal.pone.0003316

**Published:** 2008-10-02

**Authors:** Zhi-Hua Chen, Hong Pyo Kim, Frank C. Sciurba, Seon-Jin Lee, Carol Feghali-Bostwick, Donna B. Stolz, Rajiv Dhir, Rodney J. Landreneau, Mathew J. Schuchert, Samuel A. Yousem, Kiichi Nakahira, Joseph M. Pilewski, Janet S. Lee, Yingze Zhang, Stefan W. Ryter, Augustine M. K. Choi

**Affiliations:** 1 Division of Pulmonary, Allergy and Critical Care Medicine, Department of Medicine, University of Pittsburgh, Pittsburgh, Pennsylvania, United States of America; 2 Pulmonary and Critical Care Medicine, Brigham and Women's Hospital, Harvard Medical School, Boston, Massachusetts, United States of America; 3 Center for Biologic Imaging, Department of Cell Biology and Physiology, School of Medicine, University of Pittsburgh, Pittsburgh, Pennsylvania, United States of America; 4 Department of Pathology, School of Medicine, University of Pittsburgh, Pittsburgh, Pennsylvania, United States of America; 5 Department of Surgery, University of Pittsburgh, Pittsburgh, Pennsylvania, United States of America; University of Birmingham, United Kingdom

## Abstract

**Background:**

Chronic obstructive pulmonary disease (COPD) is a progressive lung disease characterized by abnormal cellular responses to cigarette smoke, resulting in tissue destruction and airflow limitation. Autophagy is a degradative process involving lysosomal turnover of cellular components, though its role in human diseases remains unclear.

**Methodology and Principal Findings:**

Increased autophagy was observed in lung tissue from COPD patients, as indicated by electron microscopic analysis, as well as by increased activation of autophagic proteins (microtubule-associated protein-1 light chain-3B, LC3B, Atg4, Atg5/12, Atg7). Cigarette smoke extract (CSE) is an established model for studying the effects of cigarette smoke exposure *in vitro*. In human pulmonary epithelial cells, exposure to CSE or histone deacetylase (HDAC) inhibitor rapidly induced autophagy. CSE decreased HDAC activity, resulting in increased binding of early growth response-1 (Egr-1) and E2F factors to the autophagy gene LC3B promoter, and increased LC3B expression. Knockdown of E2F-4 or Egr-1 inhibited CSE-induced LC3B expression. Knockdown of Egr-1 also inhibited the expression of Atg4B, a critical factor for LC3B conversion. Inhibition of autophagy by LC3B-knockdown protected epithelial cells from CSE-induced apoptosis. *Egr-1* ^−/−^ mice, which displayed basal airspace enlargement, resisted cigarette-smoke induced autophagy, apoptosis, and emphysema.

**Conclusions:**

We demonstrate a critical role for Egr-1 in promoting autophagy and apoptosis in response to cigarette smoke exposure in vitro and in vivo. The induction of autophagy at early stages of COPD progression suggests novel therapeutic targets for the treatment of cigarette smoke induced lung injury.

## Introduction

Chronic obstructive pulmonary disease (COPD) is defined as a preventable and treatable disease state characterized by airflow limitation that is not fully reversible. The airflow limitation is usually progressive and is associated with an abnormal inflammatory response of the lungs to noxious particles or gases [1]. Cigarette smoking is the most important risk factor for the development of COPD [2]. However, only 10–15% of smokers develop the disease, implying genetic factors may increase the risk in certain individuals [3]. The cellular and molecular mechanisms for COPD pathogenesis remain incompletely understood [4].

Gene expression profiling studies of human clinical COPD lung tissue have suggested candidate molecular targets for disease progression and therapeutic intervention [5]–[6]. In our recent comprehensive gene expression analyses, we found a number of genes that were differentially expressed between COPD smokers with and without lung function impairment. Among these, we identified the early growth response-1 (Egr-1) as a gene whose expression changed significantly in COPD [6]. Egr-1 is a zinc finger transcription factor classified as an immediate-early response protein, which is potentially induced by a variety of cellular stressors [7]. Egr-1 regulates the expression of many genes, including repair enzyme systems, angiogenic factors, cytokines, apoptotic factors, cell cycle factors, metabolic factors and proteases [8]. The role of Egr-1 in the pathogenesis of COPD remains incompletely understood.

Recent studies have implicated other epigenetic factors potentially involved in COPD progression, among which include histone deacetylases (HDAC)[9]–[10]. HDACs belong to a family of nuclear enzymes that modify the expression of inflammatory genes by regulating chromatin structure [10]. Acetylation of core histones leads to changes in chromatin structure that subsequently allow transcription factors and RNA polymerase II to bind to DNA and enhance gene transcription. Conversely, deacetylation of histones is generally associated with the repression of transcription. HDACs can also modulate the acetylation state and activity of major transcription factors [10]. The relationship between HDAC activity and the regulation of Egr-1 is currently unknown.

Programmed cell death mechanisms may contribute significantly to lung cell death and tissue injury following exposure to cigarette smoke, though their role in COPD pathogenesis remains unclear [11]. Recent examination of lung tissue from COPD patients reveals the presence of apoptotic cells in greater numbers than in control lungs or those from smokers without COPD [12]–[13]. Apoptotic cells include alveolar and bronchial epithelial cells as well as endothelial cells in the parenchyma. In rodents, induction of endothelial or epithelial apoptosis is accompanied by loss of pulmonary alveoli and pathological evidence of emphysematous changes [14]. Blockade of the vascular endothelial growth factor receptors caused alveolar cell apoptosis, oxidative stress and emphysema-like disease in both rats and mice [15]. This evidence suggests that apoptosis is a crucial step in emphysema and COPD.

Autophagy is a dynamic process responsible for the turnover of cellular organelles and long-lived proteins. During autophagy, cytosolic proteins and organelles (*e.g.*, mitochondria and endoplasmic reticulum) are engulfed into double-membrane bound vesicles (autophagosomes or autophagic vacuoles). The outer membrane of the autophagosome subsequently fuses with lysosomes to form autolysosomes in which the engulfed components are degraded by lysosomal hydrolases, regenerating metabolic precursors that are recycled for macromolecular synthesis and ATP generation. Autophagy is induced above basal levels in response to diverse stimuli including nutrient starvation, genotoxic agents, cytokines, and oxidative stress. This process provides an essential function in the maintenance of cellular homeostasis and adaptation to adverse environments. Under certain conditions, however, excessive autophagy may promote cell death [16]–[17]. Genes critical for the regulation of autophagy were initially discovered in yeast, though recently, extensive investigation has been done in mammalian systems since the discovery of their eukaryotic homologs [16]–[17]. The regulation and functional significance of autophagy in human tissues under physiological or pathological conditions remains incompletely understood. Furthermore, nothing is currently known of the possible role of autophagy in emphysema and COPD.

We report here for the first time that increased autophagy is associated with cigarette smoke-induced lung injury and COPD. We sought to determine the specific relationships between epigenetic factors, such as HDAC activities and Egr-1, which are modulated by cigarette smoke, and their roles in the regulation of autophagy by cigarette smoke. The current study demonstrates that cigarette smoke-dependent decreases in HDAC activity are functionally linked to the induction of autophagy. HDAC inhibition increases the expression of microtubule-associated protein-1 light chain-3B (LC3B), a key mediator of autophagy. We show that this process involves an HDAC-dependent regulation of E2F and Egr-1 transcription factors, which cooperatively regulate the LC3B promoter. Egr-1 is also essential for the regulation of Atg4B, which regulates the activation state of LC3B. We identify Egr-1 as an important regulator of lung cellular and tissue responses to cigarette smoke exposure. Our results implicate Egr-1 as a key mediator of cigarette smoke-induced autophagy and apoptosis *in vitro* and *in vivo*, and thereby demonstrate a critical role for Egr-1 in COPD pathogenesis.

## Results

### Increased autophagy in human COPD lung tissues

**Figure 1:**
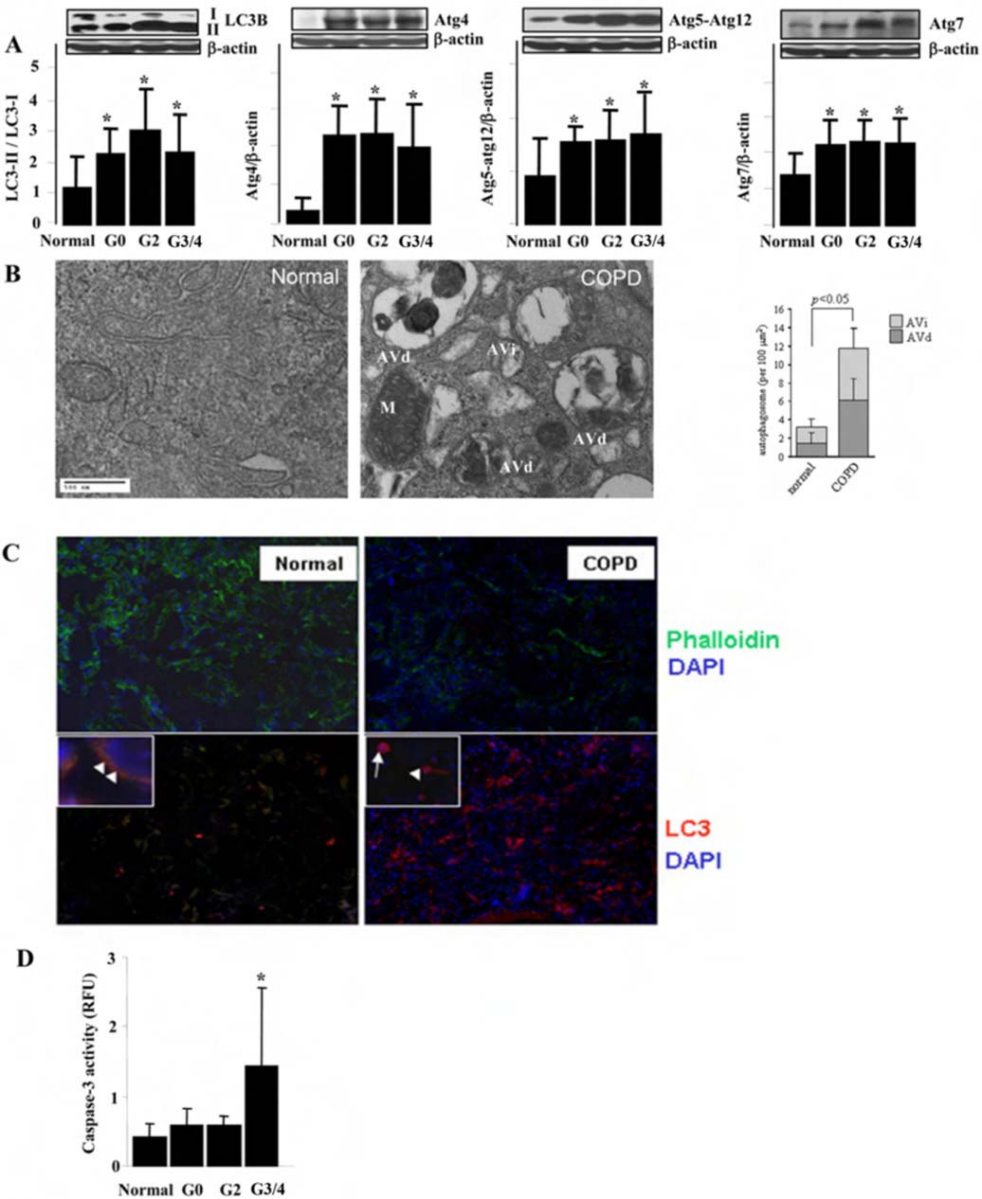
Autophagy and apoptosis in Lung Tissues from COPD Patients. (A) Western blot analysis and corresponding quantification of autophagy related proteins, LC3-II/LC3-I, Atg4, Atg5-atg12 and Atg7, in human lung tissues from normal and COPD patients. Images are representative blots of the corresponding proteins. The sample numbers analyzed by densitometry for each group are: Normal (n = 12), G0 (n = 12), G2 (n = 12), G3/4 (n = 20). β-Actin served as the standard. (B) Representative EM study of human lung sections from a nonsmoker or COPD patient. M indicates mitochondria. AVi indicates immature autophagic vacuoles (autophagosomes). AVd indicates degradative, late autophagic vacuoles (autolysosomes). (*Insert*, *Right*) Peripheral lung epithelial cells were scored for number of immature autophagic vacuole (AVi) and degradative autophagic vacuoles (AVd). The data are represented as AVi and AVd per 100 µm^2^. Data are mean scores plus standard deviation. N = 30 images representative of COPD lung, and N = 15 images representative of control, at same magnification. *P<0.05 (C) Representative immunofluorescence staining of human lung sections from normal and COPD patient. Upper two images are tissues stained with Phalloidin (green), indicating the lung alveolar structure. Lower two images are tissues stained with LC3 (red). Images are at 200× and 600× (insets). White arrow indicates the immune cells and white arrowhead indicates epithelial cells. (D) Caspase-3 activity of lung tissues from normal and various stages of COPD patients (n = 10). (B, D) Data represent mean+/−S.D. *indicates P<0.05, ** indicates P<0.01.

To investigate the role of autophagy in the pathogenesis of COPD, lung tissue sections from control or COPD patients were analyzed for the expression of autophagy proteins. In mammals, the conversion of LC3B from LC3B-I (free form) to LC3B-II (membrane-bound form) is regarded as a key step in the induction of autophagy [16]–[17]. The ratio of LC3B-II/LC3B-I, as well as the expression of Atg4B, Atg5-Atg12 and Atg7 were significantly increased in COPD lung (Fig. 1A). Electron microscopy (EM), a gold-standard method for determination of autophagy, revealed that autophagic vacuoles (autophagosomes/autolysosomes) were dramatically increased in COPD lung tissues, whereas little vacuole formation was evident in control tissues (Fig. 1B). Immunofluorescence staining indicated that the expression of LC3B was significantly increased in COPD lung tissues (Fig. 1C). Immunohistochemical staining of COPD lung tissue indicated that LC3B expression was localized to epithelial cells and macrophages (*data not shown*). Examination of various stages of COPD, according to the Global Initiative for Obstructive Lung Disease (GOLD) for classifying disease severity in COPD, revealed elevations of these markers at GOLD0 which were sustained throughout the disease progression (GOLD2-4), (Fig. 1A), suggesting that autophagy represents a response to cigarette smoke exposure, as well as an early event in the progression of emphysema. Lungs from patients with α-1 anti-trypsin deficiency (α1AT), the genetic cause of emphysema, also showed significant induction of autophagy (Table 1). However, lungs from patients with other pulmonary diseases, including idiopathic pulmonary fibrosis, cystic fibrosis, sarcoidosis, and systemic sclerosis, showed no significant elevation of autophagy (Table 1).

**Table 1:** Relative expression of autophagic proteins in human lung tissues.

	Normal	GOLD0	GOLD2	GOLD3/4	CF	α1AT	Sarc	SSc	IPF
	(n = 12)	(n = 12)	(n = 12)	(n = 20)	(n = 10)	(n = 7)	(n = 4)	(n = 6)	(n = 18)
**LC3-II/LC3-I**	5.75±5.24	11.10±4.21#	15.13±6.67#	11.40±6.30#	2.61±1.63	12.21±4.88#	3.25±1.69	1.51±1.47	1.90±1.40
**Atg4**	0.067±0.064	0.47±0.16*	0.48±0.18*	0.40±0.24*	0.12±0.12	0.21±0.15	0.18±0.16	0.22±0.18	0.35±0.19#
**Atg5-atg12**	0.40±0.29	0.61±0.13#	0.63±0.23#	0.67±0.32#	0.43±0.20	0.26±0.08	0.40±0.17	0.43±0.14	0.46±0.13
**Atg7**	0.32±0.13	0.45±0.14#	0.47±0.12#	0.46±0.14#	0.31±0.19	0.49±0.11#	0.34±0.11	0.31±0.21	0.38±0.18

Table 1 shows the quantified Western blot analysis of Autophagy-related proteins, LC3-II/LC3-I, Atg4, Atg5-atg12 and Atg7, in human lung tissues from normal patients, from various stages of COPD (GOLD0, GOLD2, GOLD3/4) and from patients with various representative lung diseases, including cystic fibrosis (CF), alpha-1 anti-trypsin deficiency (α1-AT), sarcoidosis (SARC), systemic sclerosis (SSc), and interstitial pulmonary fibrosis (IPF). The sample numbers analyzed for each group are: Normal (n = 12), G0 (n = 12), G2 (n = 12), G3/4 (n = 20), IPF (n = 18), CF (n = 10), Sarc (n = 4), SSc (n = 6) and α1AT (n = 7). Data represent mean±SD. Atg4, Atg5-atg12 and Atg7 data were normalized to β-actin.

* p<0.01 compared with normal group.

# p<0.05 compared with normal group.

### Apoptosis in COPD lung tissues

Autophagy represents a cellular adaptive mechanism that promotes survival under various stress conditions including nutrient deprivation [16]–[17]. However, excessive autophagy can influence several forms of cell death, including caspase-independent cell death and apoptosis [18]–[24]. We therefore examined apoptotic markers in COPD patients, as increasing evidence suggests that apoptosis plays a pivotal role in the pathogenesis of COPD [11]–[15]. Analysis of lung tissues from different stages of COPD revealed that Caspase-3 activity was only significantly elevated in GOLD3/4 (Fig. 1D). These data suggest that apoptosis occurs later than autophagy during the development of emphysema and COPD.

### Cigarette smoke exposure increases epithelial cell autophagy

**Figure 2:**
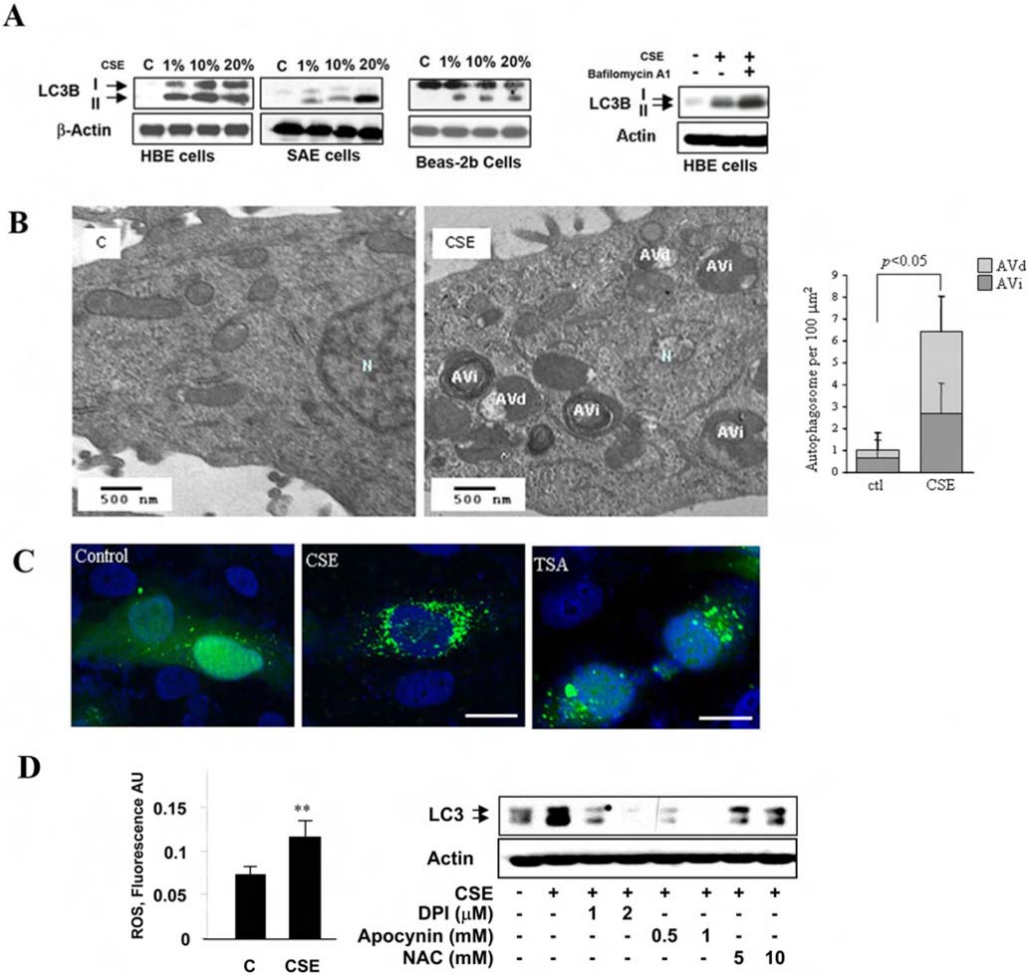
Increased Autophagy by CSE in Pulmonary Epithelial Cells. (A) Western blot analysis of LC3 in CSE-treated HBE, SAE, and Beas-2B cells. Cells were treated with various concentrations of CSE for 24 h. Far right panel shows that bafilomycin A1 enhanced the CSE-induced LC3 expression in HBE cells. Cells were pretreated with 200 nM Bafilomycin A1 for 30 min and followed by the exposure to 30% CSE for an additional 24 h. (B) Formation of autophagic vacuoles in HBE cells treated with 1% CSE for 4 h. N indicates nuclei. AVi, immature autophagic vacuoles (autophagosomes), AVd, degradative, late autophagic vacuoles (autolysosomes). (*Right*) EM images corresponding to HBE cell exposed to 10% CSE for 24 h scored for number of immature autophagic vacuole (AVi) and degradative autophagic vacuoles (AVd). The data are represented as AVi and AVd per 100 µm^2^. Data are mean scores plus standard deviation. N = 15 images representative of CSE treated cells, and N = 10 images representative of control cells, at same magnification. *P<0.05 (C) Representative images of the punctuated GFP-LC3 in Beas-2B cells treated with 5% CSE or 50 nM TSA for 24 h. White bar indicates 10 µm. (D) Increased ROS accumulation in CSE-treated Beas-2B cells. Cells were exposed to 20% CSE for 1 h. (*Right*) CSE-induced LC3 expression was attenuated by diphenyliodonium (DPI), Apocynin or N-acetyl-L-cysteine (NAC). Beas-2B cells were pretreated with the antioxidant at the indicated concentrations for 30 min and followed by the exposure to 30% CSE for an additional 24 h. Data represent mean+/−S.D. of three independent experiments. ** indicates P<0.01.

Although CSE exposure cannot replicate the complex effects of long term cigarette smoking in an *in vivo* system, it facilitates the testing of human and mouse lung derived cells for responses to the ingredients contained in cigarette smoke. We studied the induction of autophagy by CSE in a human pulmonary cell line and in primary cell cultures. Biochemical studies revealed a dose-dependent induction of LC3B-II/LC3B-I ratio by CSE in human bronchial epithelial (HBE) cells grown in air-liquid interface cultures, as well as in cultured SAE and Beas-2B cells (Fig. 2A). While some variations were detected in basal LC3B-I levels among the cell lines, all lines displayed increased LC3B-II levels with 1% CSE exposure. Furthermore, the induction of LC3B-II levels by CSE in HBE cells was enhanced by bafilomycin A1 (Fig. 2A), a compound that prevents the maturation of autophagic vacuoles by inhibiting autophagosome-lysosome fusion [25]. EM analysis of HBE cells revealed dramatic induction of autophagic vacuoles by CSE as early as 4 h (Fig. 2B). GFP-LC3 fluorescence was used to monitor autophagy in Beas-2B cells transiently transfected with an expression vector for GFP-LC3 [26]. The formation of GFP-LC3-labeled puncta, representing association of LC3B-II with autophagosomes (27), was extensively induced in cells exposed to CSE (Fig. 2C). We examined whether the induction of autophagy by CSE was related to altered cellular redox homeostasis. We have observed that CSE increased ROS levels in Beas-2B cells. CSE induced autophagy was significantly attenuated in the presence of NADPH: oxidase inhibitors, diphenylene iodonium or apocynin, or the antioxidant, N-acetyl-L-cysteine (Fig. 2D).

### Relationship of autophagy and apoptosis in cigarette smoke exposure

**Figure 3:**
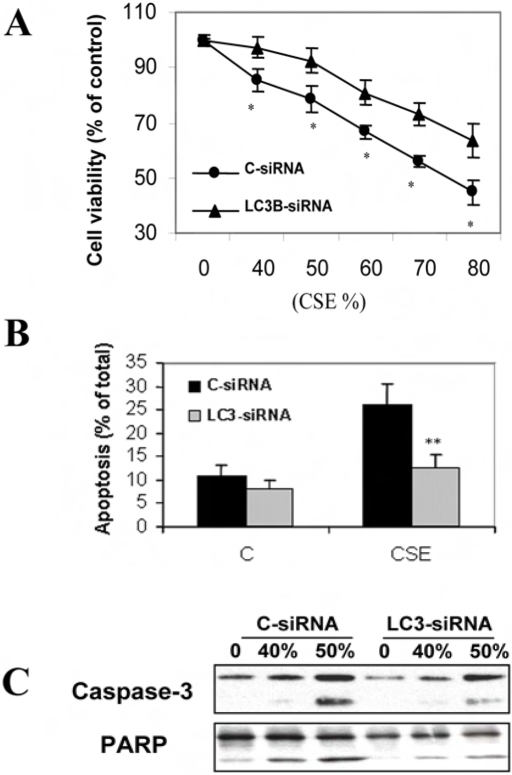
Relationship of autophagy to epithelial cell death. (A–C) Beas-2B cells were pretreated with C-siRNA or LC3-siRNA for 48 h followed by exposure to the indicated concentrations of CSE for an additional 24 h. Cells were then subjected to MTT assay (A), Annexin-V staining (B) or Western blot analysis (C). (A) LC3-siRNA attenuated CSE-induced cytotoxicity in Beas-2B cells. (B) LC3-siRNA inhibited CSE-induced apoptosis in Beas-2B cells. (C) LC3-siRNA attenuated CSE-induced caspase-3 activation and PARP cleavage. (A, B) Data represent mean±SD, (n = 3, * indicates *P*<0.05 and ** indicates *P*<0.01 compared with the corresponding control data).

Increasing evidence suggests crosstalk between apoptosis and autophagy, as several apoptosis-related factors are critically involved in autophagy [21]–[23]. Inhibition of apoptosis may trigger autophagy, [18]–[19] whereas inhibition of autophagy may either induce or attenuate apoptosis [22]–[24]. We therefore examined the functional significance of autophagy in relationship to cell death caused by cigarette smoke. Inhibition of LC3B by siRNA attenuated CSE-induced cytotoxicity in Beas-2B cells (Fig. 3A). In LC3B-siRNA treated cells, CSE-induced cell apoptosis was also significantly inhibited, as revealed by the decreased number of apoptotic cells as well as reduced caspase-3 activation and poly (ADP-ribose) polymerase (PARP) cleavage (Figs. 3B and 3C).

### Decreased HDAC activity regulates autophagy in response to cigarette smoke exposure

**Figure 4:**
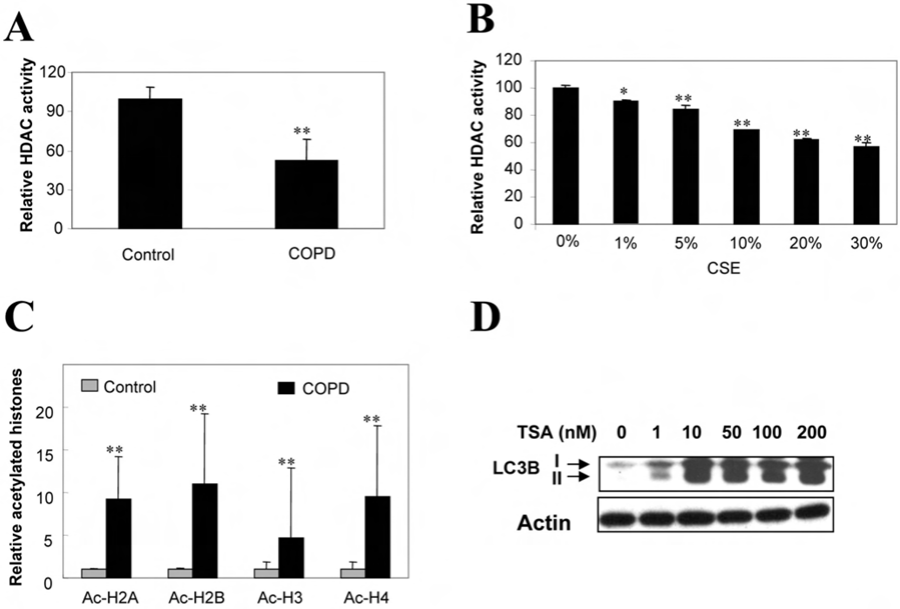
Role of HDAC Activity in COPD and Autophagy. (A) Decreased HDAC activity in lung tissues from COPD patients (n = 5). (B) CSE-dependent reduction of HDAC activity in Beas-2B cells. Cells were treated with various concentrations of CSE for 24 h and subjected for analysis of HDAC activity. (C) Western blot analysis of acetylated histones in human lung tissues. (D) Induction of LC3 by TSA in Beas-2B cells. Cells were treated with the indicated concentrations of TSA for 24 h. (B–C) Data represent mean+/−S.D. of three independent experiments. *indicates P<0.05, ** indicates P<0.01.

Recent studies have shown decreased HDAC activity as well as HDAC protein levels in COPD and in cells exposed to cigarette smoke extract (CSE) [9], [28]–[29]. Since the inhibition of HDAC activity can induce both apoptotic and autophagic cell death in vitro [30], we hypothesized that decreased HDAC activity in COPD may also trigger autophagy. Consistently, we found that HDAC activity was significantly reduced in COPD (Fig. 4A), and dose-dependently decreased by CSE in Beas-2B cells (Fig. 4B). In human COPD, acetylation of histones H2A, H2B, H3, and H4 was increased (Fig. 4C). Tricostatin-A (TSA), the inhibitor of HDAC, significantly induced autophagy, as revealed by increased LC3B expression (Fig. 4D) and GFP-LC3 puncta, at pharmacological concentrations (Fig. 2C).

### Regulation of LC3B by Egr-1

**Figure 5:**
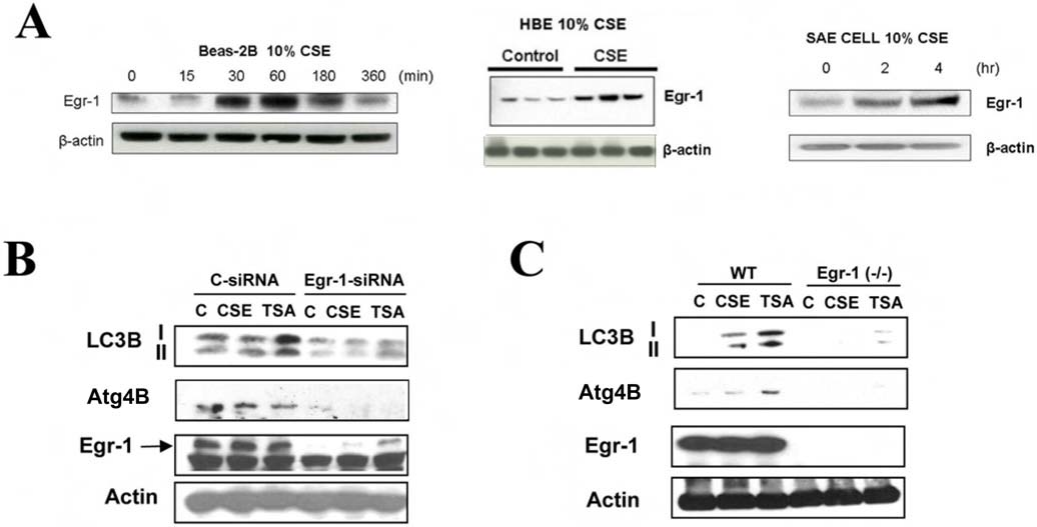
Expression of LC3B requires Egr-1. (A) Beas-2B, HBE, or SAE cells were treated with 10% CSE, for the times indicated and assayed for the expression of Egr-1 by Western immunoblot analysis. Images are representative of 3 independent experiments. (B) Beas-2B cells were pretreated with control siRNA (C-siRNA), or Egr-1 siRNA for 48 h, followed by the treatment with 20% CSE or 100 nM TSA for an additional 24 h. Cells were then harvested for Western blot analysis. β-Actin served as the standard. (C) Lung fibroblasts derived from wild type C57BL/6 or Egr-1^−/−^ mice were treated with 20% CSE or 100 nM TSA for 24 h. Lysates were analyzed by Western immunoblotting for LC3, or Egr-1. β-Actin served as the standard.

Our laboratory has previously performed comprehensive gene expression analyses which have indicated that the transcriptional regulator early growth response-1 (Egr-1) represents one of the major genes that are significantly upregulated in COPD lung tissue [6]. We have also previously shown that Egr-1 protein expression is increased in normal human lung fibroblasts cells after exposure to CSE in a dose- and time-dependent manner [6]. We therefore tested whether a similar response could be observed in pulmonary epithelial cells. Treatment of Beas-2B cells with CSE (10%) induced the time-dependent expression of Egr-1 protein, with an apparent maximum at 60 min of exposure time (Fig. 5A). Similar results were observed in HBE and SAE cells (Fig. 5A). We hypothesized that Egr-1 may play a role in the regulation of autophagy genes such as *LC3B* and *Atg4B*. Genetic inhibition of Egr-1 (Fig. 5B) by siRNA significantly attenuated CSE- and TSA-inducible LC3B and Atg4B expression. Lung fibroblasts derived from *Egr-1* ^−/−^ mice also showed remarkably attenuated expression of LC3B and Atg4B (Fig. 5C) in response to CSE or TSA.

**Figure 6:**
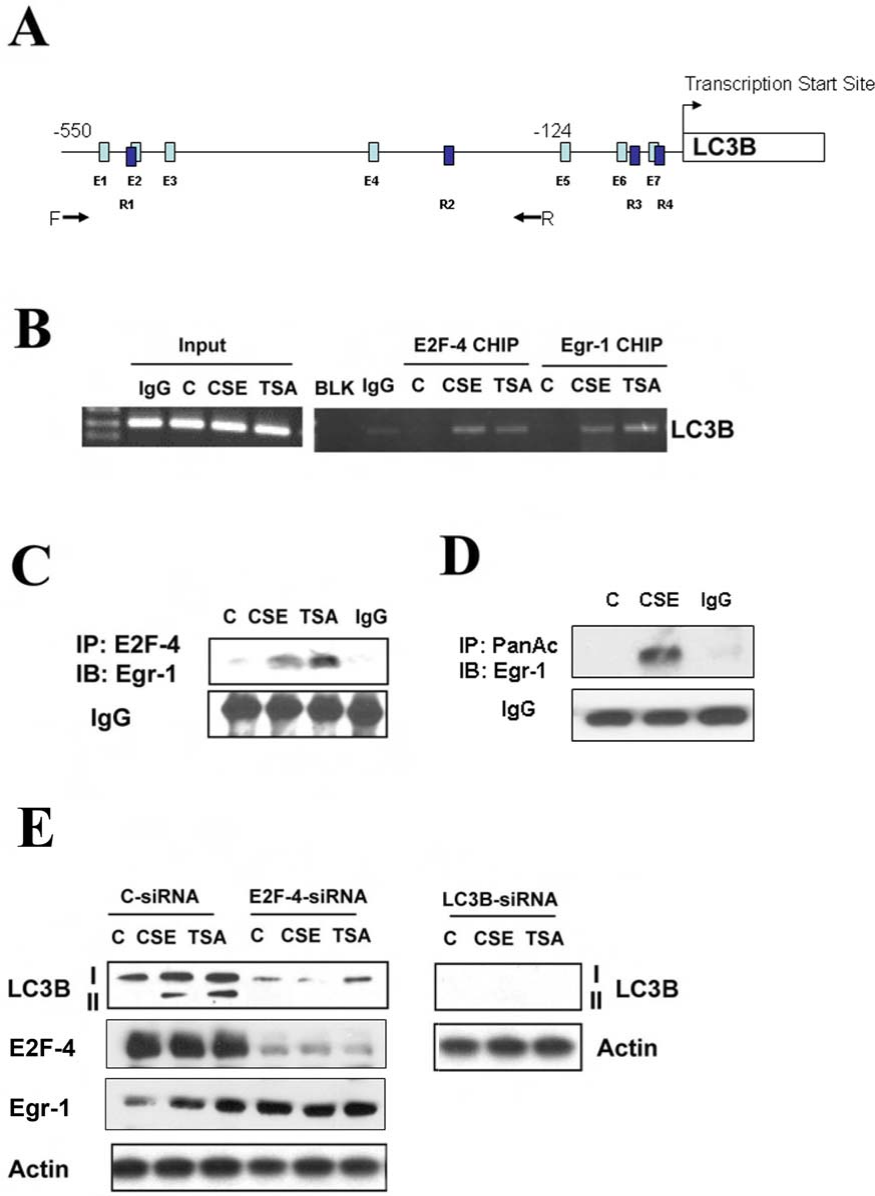
Regulation of LC3B by Egr-1 and E2F-4. (A) Putative Egr1 and E2F4 binding sites in the LC3b promoter. PCR primer locations for the CHIP analysis are labeled by arrows. Putative E2F4 (E1 to E7) and Egr1 (R1 to R4) binding sites are indicated as light and dark blue bars, respectively. The nucleotide locations for putative E2F1 and Egr1 sites are shown in Figure S1. (B) Increased DNA binding of E2F-4 and Egr-1 to the LC3B promoter by CSE and TSA. Beas-2B cells were treated with 20% CSE or 100 nM TSA for 1 h and subjected to CHIP assay as described in Methods. Chromatin samples were immunoprecipitated with anti-E2F-4 or anti-Egr-1 and evaluated for factor binding to the LC3B promoter region. (C) Beas-2B cells were treated with 20% CSE or 100 nM TSA for 1 h. Lysates were immnuoprecipitated (IP) with anti-E2F-4 and immunoblotted (IB) with anti-Egr-1. IgG served as the standard. CSE and TSA increased E2F-4 and Egr-1 complex formation. (D) Beas-2B cells were treated with 20% CSE for 24 h. Lysates were immunoprecipitated (IP) with anti-pan acetyl antibody and immunoblotted (IB) with anti-Egr-1. IgG served as the standard. CSE increased acetylation of Egr-1. (E) Effect of E2F-4 on the CSE- and TSA-induced LC3B expression. Beas-2B cells were pretreated with control siRNA (C-siRNA), E2F-4-siRNA, LC3B siRNA for 48 h, followed by the treatment with 20% CSE or 100 nM TSA for an additional 24 h. Cells were then harvested for Western blot analysis. β-Actin served as the standard.

Since little is known about the transcriptional regulation of the *LC3B* gene, we sought to identify possible regulatory factors of this autophagy gene. Genetic analysis of the promoter region of *LC3B* revealed several candidate transcription factor binding sites, including binding sites for Egr-1 and E2F (see Fig 6A and Materials and Methods). Chromatin immunoprecipitation assays revealed that Egr-1 and E2F-4 were rapidly induced to bind the *LC3B* promoter under stimulation by CSE or TSA in Beas-2B cells (Fig. 6B).

HDACs form complexes with and thereby regulate the activity of members of the E2F family of transcription factors, important regulators of cell cycle progression and proliferation [31]–[33]. We therefore examined a possible link between HDAC inhibition and the potential regulation of *LC3B* by E2F and Egr-1 factors. E2F-4 formed a complex with Egr-1 following cellular stimulation with CSE or the HDAC inhibitor TSA (Fig. 6C). Consistent with our observations of reduced HDAC activity in CSE-treated Beas-2B cells, we have observed increased acetylation of Egr-1 protein in response to CSE treatment (Fig. 6D). Genetic inhibition of E2F-4 by siRNA significantly attenuated CSE- and TSA-inducible LC3B expression (Fig. 6E), whereas did not alter the expression of Egr-1 (Fig. 6E).

### Role of Egr-1 in the regulation of autophagy, apoptosis, and emphysema in response to cigarette smoke exposure in vivo

**Figure 7:**
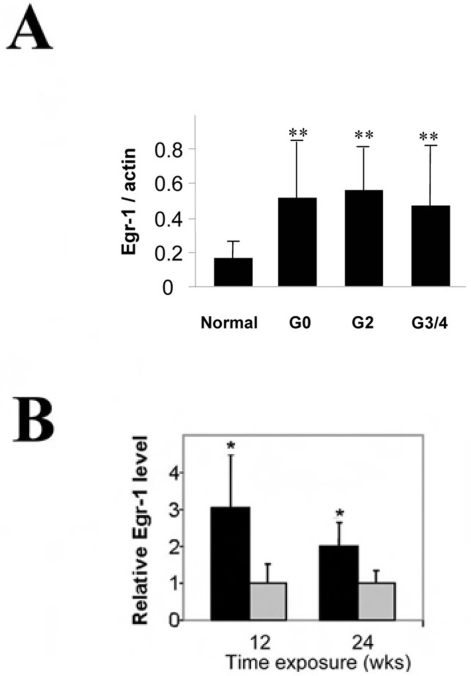
Expression of Egr-1 in vitro and in vivo in response to cigarette smoke. (A) Lung tissue samples from various stages of COPD (GOLD0 to GOLD3/4) were analyzed for expression of Egr-1 in lung tissue by Western immunoblot analysis, and normalized to β-actin expression. (B) C57BL/6 were exposed to chronic cigarette smoke exposure (black bars) or air (grey bars) for 12 or 24 weeks and Egr-1 expression in the lung was determined by Western immunoblot analysis. β-Actin served as the standard. (n = 12). *indicates P<0.05, **indicates P<0.01. N.S., not significant.

To assess the role of Egr-1 in human disease, we also examined Egr-1 protein expression in COPD human lung tissue specimens. Egr-1 protein expression was elevated at all stages of COPD relative to normal controls (Fig. 7A). The expression of Egr-1 was increased in C57Bl/6 mouse lung tissue after 12 or 24 weeks of chronic cigarette smoke exposure relative to air-exposed controls (Fig. 7B).

**Figure 8:**
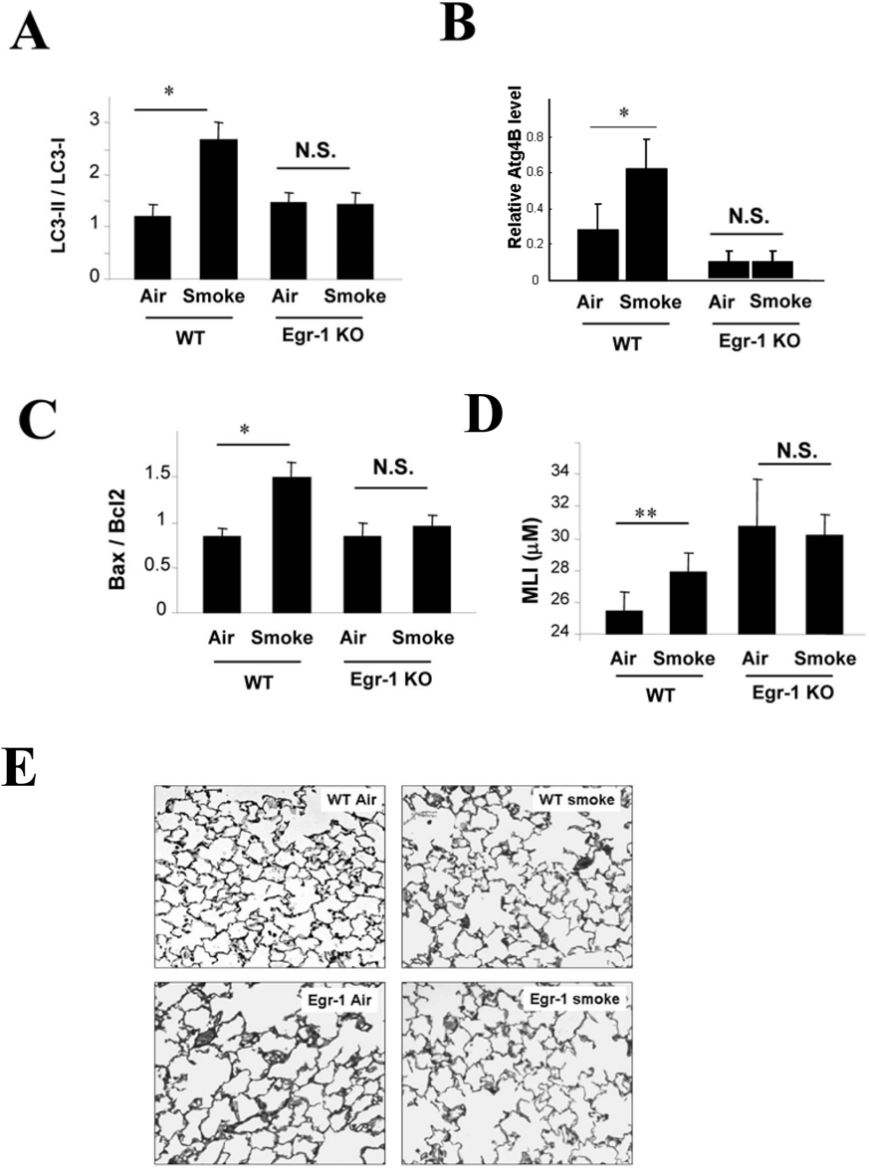
Role of Egr-1 in the regulation of autophagy, apoptosis, and emphysema *in vivo* during chronic cigarette smoke exposure. (A–C) Wild type C57BL/6 or *Egr-1* ^−/−^ mice were exposed to chronic cigarette smoke exposure for 6 months (each group n = 5). Lung tissue homogenates were analyzed for (A) expression of LC3, (B) expression of Atg4B, (C) Bax/Bcl_2_ ratio. Lung tissue was evaluated for emphysema/airspace enlargement and mean linear intercept (D) and H+E staining (E).

*Egr-1* ^−/−^ mice displayed attenuated activation of LC3B (Fig. 8A), Atg4B expression (Fig. 8B) and apoptotic factors (*e.g.*, Bax/Bcl-2 ratio)(Fig. 8C) in response to prolonged (6 mo) cigarette smoke exposure relative to wild-type counterparts. *Egr-1* ^−/−^ mice basally exhibited airspace enlargement as assessed by mean linear intercept (MLI) [34], relative to corresponding wild-type controls, whose magnitude exceeded that of cigarette smoke-exposed wild-type mice. Chronic smoke exposure did not modulate airspace enlargement as assessed by MLI and H+E staining in *Egr-1* ^−/−^ mice (Fig. 8D and E). These results indicate that *Egr-1* ^−/−^ mice, which displayed basal airspace enlargement, resisted cigarette-smoke induced autophagy and apoptosis, as well as further changes in airspace enlargement as assessed by MLI.

## Discussion

Here, we provide the first evidence of cigarette smoke-induced autophagy in human COPD, in pulmonary epithelial cells, and in mice exposed to chronic cigarette smoke. We also examined the relationship between autophagy and apoptosis in response to cigarette smoke. We have observed that blockage of autophagy by LC3B-siRNA attenuated CSE-induced epithelial cell apoptosis and consequently increased cell viability, suggesting that autophagy functioned as a mediator of apoptosis in this model. Consistently, autophagy was induced at very low CSE concentrations (as low as 1%) and at earlier times (as early as 4 h)(Fig. 2B). In COPD patients, as early as GOLD0, there was dramatic increase of autophagy and autophagic proteins (Figs. 1A–C) which was evident throughout the disease progression (up to GOLD4), whereas caspase-3 activation occurred only at later stages (Fig. 1D). These findings, taken together, suggest that autophagy is an essential and early response to cigarette smoke which mediates apoptosis and eventually promotes cell death, thereby potentially playing an important role in COPD pathogenesis.

Some limitations exist in the use of CSE to study *in vitro* responses to cigarette smoke, including exclusion of some volatile components present in cigarette smoke, and variations in the preparation method in between individual laboratories. Nevertheless CSE remains a useful model of *in vitro* smoke exposure that has the potential to uncover important biological pathways [35]. It should be mentioned that the CSE concentrations used in our present study appear to be higher than other previous reports by other investigators. In previous work, CSE is typically prepared in serum free medium, and used in cultures preconditioned with serum free media. In our present study, we compose our CSE in complete culture medium to avoid induction of autophagy through the serum starvation pathway. Higher CSE concentrations are apparently required for apoptosis and cell death in complete medium relative to serum free media.

Epigenetic factors have recently come into focus as possible mechanisms underlying susceptibility to COPD in certain individuals [36]. Surgically resected lung tissue from nonsmokers or GOLD0-4 smokers, showed significant decreases of HDAC activity in COPD lung tissue relative to nonsmokers whose magnitude correlated with disease severity and inflammatory gene expression [9]. In addition to lung tissue, HDAC activity was also decreased in alveolar macrophages from bronchoalveolar lavage samples of COPD patients [9]. Furthermore, the expression of HDAC2 mRNA and protein in peripheral lung tissue and bronchoalveolar lavage macrophages, declined as a function of disease progression [9]. Consistent with these prior studies, we have observed decreased HDAC activity in human COPD lung tissue. Furthermore, we show that the acetylation of major histones was increased in COPD lung tissue. Exposure to CSE dose-dependently decreased HDAC activity in pulmonary epithelial cells *in vitro* (Fig. 4B). While HDAC activity is generally associated with transcriptional repression, through the deacetylation of histones, HDACs may also modulate the acetylation state of major transcription factors, and thus have complex effects on gene regulation. Previously, these activities have been linked to the regulation of pro-inflammatory gene expression in chronic lung diseases [36]. Here, we have suggested a link between HDAC modulation and the regulation of autophagy, as the HDAC inhibitor TSA (Figs. 4D and 2C) and CSE exposure (Figs. 2A–C) induced autophagy *in vitro* (formation of LC3 puncta, and increased LC3B expression and conversion). Decreases in HDAC led to increases in complex formation between the transcription factor Egr-1 and its coactivator E2F-4, both which displayed increased binding at the LC3B promoter (Fig. 6). While HDACs have previously been shown to regulate E2F factors [31]–[33], we demonstrate that Egr-1 is also regulated by HDAC, such that increased acetylation of the protein was detected with CSE treatment. Our current findings strongly demonstrated a central role of E2F-4 and Egr-1 in regulation of LC3B expression, and thereby also suggest novel targets for regulating autophagy in mammalian systems.

We also demonstrate that Egr-1 is essential for the upregulation of Atg4B by cigarette smoke (Fig 5B and C). Atg4B is essential in the cleavage of pro-LC3B to LC3B-I, as a prerequisite for the conjugation of LC3B-I with phosphatydylethanolamine by Atg3/Atg7 ligases. Atg4 plays a dual role in that it also catalyzes the deconjugation of LC3B-II during late stage autophagy [37]. Therefore we conclude that Egr-1 directly regulates LC3B expression by transcriptional regulation of the gene, and also indirectly regulates LC3B conversion through regulation of Atg4B.

Egr-1 is expressed in the mouse lung in response to cigarette smoke exposure and also *in vitro* in response to CSE exposure (Fig 5A and 7B). We have shown here that elevated Egr-1 expression is also evident in human clinical samples of COPD at all stages of disease progression (Fig. 7A). Recent studies from this laboratory have revealed that Egr-1 may play a role in cigarette smoke induced lung pathophysiology by regulating the expression of matrix metalloproteinase-2, a candidate molecule in emphysema development [6]. We have further shown here that *Egr-1* ^−/−^ mice are resistant to the pro-apoptotic and pro-autophagic effects of smoke inhalation, as well as cigarette smoke-induced airspace enlargement as assessed by MLI. However, *Egr-1* ^−/−^ mice basally exhibited airspace enlargement, relative to corresponding wild-type controls (Fig. 8). Since chronic cigarette smoke exposure did not promote further increases in airspace enlargement in *Egr-1* ^−/−^ mice, these mice were apparently resistant to smoke-induced injury. While Egr-1 plays an important role in mediating cigarette smoke-induced autophagy and apoptosis *in vitro*, this factor, as suggested by previous studies [38], exerts a dominant role in lung homeostasis and growth *in vivo*. Thus, *Egr-1* ^−/−^ mice reach a critical threshold of airspace enlargement under basal conditions such that further histologic changes induced by cigarette smoke are not evident *in vivo*. Examination of the role of Egr-1 in lung development may be worthy of further investigation.

To demonstrate the pivotal role of autophagy in COPD pathogenesis, further experimentation with autophagy gene knockout mice may be warranted. However, to date, no such mice have been used for *in vivo* models of lung disease. *Atg5* ^−/−^ and *Beclin-1* ^−/−^ mice are embryonic lethal [39]–[40]. *Beclin-1* ^+/−^ mice easily develop carcinoma [40], limiting their usefulness as a model. *LC3B* ^−/−^ knockout mice have been developed recently [41], but autophagy can still occur in such mice by compensatory mechanisms. Nevertheless, the use of autophagy-deficient mice will be the focus of future investigation.

In conclusion, we provide the first evidence that autophagy occurs as an early event in COPD progression, and in epithelial cell responses to cigarette smoke exposure. Inhibition of autophagy protected against apoptosis induced by cigarette smoke. While autophagy has been recently studied in experimental models of human disease [42], very few studies have examined this process in clinical samples of human diseased tissue. Recent observations in inflammatory bowel disease suggest that genetic polymorphisms in autophagy genes may be linked to the progression of this disease [42]. Specifically, a small nucleotide polymorphism of the *atg-16L1* gene has been associated with increased susceptibility to Crohn's disease [43]–[44].

**Figure 9:**
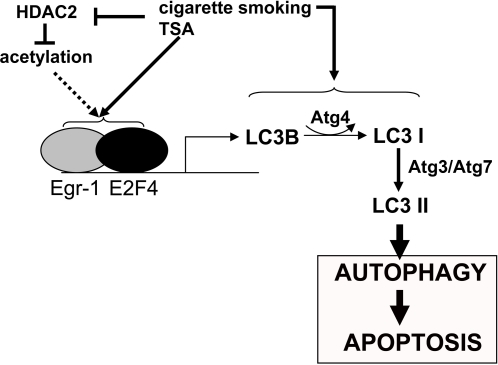
Epigenetic regulation of autophagy by cigarette smoking. The scheme shows the proposed pathway for epigenetic regulation of autophagy by cigarette smoke exposure. Suppression of HDAC activity leads to the increased transcription of the LC3B promoter by E2F/Egr-1 factors.

We propose a mechanistic pathway for the early events in the development of COPD (Fig. 9). We show that decreased HDAC activity in COPD [9], is linked with the induction of autophagy. The activation of E2F-4 and Egr-1 transcription factors by HDAC inhibition induced the expression of the autophagic regulator LC3B. Furthermore we demonstrate that Egr-1 plays a critical role in cigarette smoke-induced autophagy and apoptosis.

## Materials and Methods

### Patients

All human lung tissues were obtained from either lung transplantation explant tissues or from lung resection from thoracic surgical cases. These lung sections are processed immediately after obtaining from thoracic surgeons (co-authors Drs. Rodney J. Landreneau, Mathew J. Schuchert) by the Tissue Core (co-author Dr. Joe Pilewski, director of Tissue Core). The isolated lung tissues are snap frozen and stored in −80°C freezers, and stored at the core facility at the Thoracic Center of Excellence (COE) in Anatomic Pathology at the University of Pittsburgh (directed by Drs. Samuel Yousem and Rajiv Dhir, both co-authors of the study) who work closely with Dr. Frank Sciurba (Director of Emphysema Research Center, and co-author) at the University of Pittsburgh for clinical and phenotypic data. This described state of the art tissue sampling infrastructure has been immensely successful in obtaining the highest quality lung tissues for RNA and protein analyses.

We followed the guidelines of the Global Initiative for Obstructive Lung Disease for classifying disease severity in COPD [45]. The smoking history of the GOLD 3/4 group was an average 56 pack years; the majority were ex smokers. The smoking history of the GOLD 0/2 group was an average of 50 pack years; >60% were ex smokers. The normal lung was obtained from non-smokers.

### Animals

All animals were housed in accordance with guidelines from the American Association for Laboratory Animal Care. The Animal Care and Use Committee of the University of Pittsburgh approved the protocols. C57/BL6 mice (Jackson Laboratories, Bar Harbor, ME) or Egr-1 deficient (*egr-1* ^−/−^) mice (Taconic, Hudson, NY) were exposed to cigarette smoke (CS) or filtered air under identical conditions beginning at 8 wks of age. Mice were exposed 5 d/week for 24 weeks.

### In vivo cigarette smoke exposure

Total body cigarette smoke exposure was performed in a stainless steel chamber (71 cm×61 cm×61 cm) using a smoking machine (Model TE-10, Teague Enterprises). The smoking machine puffs each 1R3F cigarette for 2 seconds, for a total of 9 puffs prior to ejection, at a flow rate of 1.05 l/min, providing a standard puff of 35 cm^3^. The smoke machine was adjusted to deliver 5 cigarettes at one time. Mice were exposed 5 d/week for 24 weeks. The chamber atmosphere was periodically measured for total particulate matter concentrations of 100–120 mg/m^3^. Carboxyhemoglobin levels in the C57/BL6 strains of mice after two weeks of cigarette exposure were typically less than 8% immediately following exposure.

### Lung morphometry

Mean linear intercept measurements were obtained using modified Image J software available from the National Institute of Health website (http://rsb.info.nih.gov/ij/). Immediately following necropsy, the right lung was inflated by gravity with 4% paraformaldehyde and held at a pressure of 30 cmH_2_0 for 15 minutes. The left lung was removed and immediately stored in liquid nitrogen for subsequent protein studies.

The right lung was gently dissected from the thorax and placed in 4% paraformaldehyde for up to 8 h. The samples were cut para-sagitally and embedded in paraffin. Modified Gills staining was performed and twelve random 1300×1030 pixel images were acquired digitally for each lung sample using a light microscope (Zeiss Axiophot, Carl Zeiss MicroImaging, Thornwood, NY) equipped with a digital camera (Zeiss Axiocam HRc, Carl Zeiss MicroImaging, Thornwood, NY) at 200× magnification. Large airways, blood vessels, and other non-alveolar structures were manually removed from the images. The Image J software program automatically thresholded the images, and a Median filter, set to a 2 pixel radius, was run to smooth the image edges. The program laid a line grid comprised of 1353 lines with each line measuring 21 pixels over the individual images. The software then counted the number of lines that ended on or intercepted alveolar tissue. These data were used to calculate the volume of air, the volume of tissue, the surface area, the surface area to tissue volume ratio. The mean linear intercept, which assesses the degree of alveolar airspace enlargement, was calculated using the equation: (4/surface area to volume tissue ratio) according to methods adapted from Dunhill [34]. The mean linear intercept increases with increasing airspace enlargement.

### Cell culture

Human lung epithelial Beas-2B cells were maintained in DMEM containing 10% fetal bovine serum and antibiotics. The primary human small airway epithelial (SAE) cells were obtained from American Type Culture Collection (ATCC, Manassas, VA) and were cultured according to the ATCC prescription. The primary human bronchial epithelial (HBE) cells were cultured in air-liquid interface from excess pathological tissue following lung transplantation and organ donation under a protocol approved by the University of Pittsburgh Investigational Review Board, as previously described [46]. For preparation of cigarette smoke extract (CSE), Kentucky 1R3F research-reference filtered cigarettes (The Tobacco Research Institute, University of Kentucky, Lexington, KY) were smoked using a peristaltic pump (VWR International). Prior to the experiments, the filters were cut from the cigarettes. Each cigarette was smoked in 6 minutes with a 17 mm butt remaining. Four cigarettes were bubbled through 40 ml of cell growth medium, and this solution, regarded as 100% strength CSE, was adjusted to a pH of 7.45 and used within 15 minutes after preparation.

Cell viability was determined by the conventional 3-(4,5-Dimethylthiazol-2-yl)-2,5-diphenyl tetrazolium bromide (MTT) assay and the apoptotic cell death was determined by Annexin V staining.

### Transmission electron microscopy and fluorescence microscopy

Lung tissue sections were fixed in formalin and embedded in paraffin. Cells were fixed in 2.5% glutaraldehyde in PBS after experimental manipulations. These tissues or cells were photographed using a JEOL JEM 1210 transmission electron microscope (JEOL, Peabody, MA) at 80 or 60 kV onto electron microscope film (ESTAR thick base; Kodak, Rochester, NY) and printed onto photographic paper. To examine the distribution of GFP-LC3B, cells were observed under a fluorescence microscope and digital images were acquired for analysis (SPOT, Diagnostic Instruments, Inc.).

### Immunofluorescence staining

Formalin-fixed, paraffin-embedded lung tissue sections were subject to immunofluoresence staining with anti-LC3. Samples were viewed with an Olympus Fluoview 300 Confocal Laser Scanning head with an Olympus IX70 inverted microscope. For analyzing reactive oxygen species (ROS), the cells were preincubated with 10 µM CM-H2DCFDA (Invitrogen) for 30 min, followed by incubation with CSE. The fluorescence of oxidized DCFDA in cell lysates, was measured on an Aminco Bowman series-2 spectrofluorometer at 490/530 nm ex/em.

### Small interfering RNA (siRNA) and GFP-LC3 transfection

Human E2F-4-siRNA was purchased from Santa Cruz Biotechnology (Santa Cruz, CA). Human LC3B-siRNA was designed and manufactured by Dharmacon Research (Layfayette, CO). The siRNA was transfected into cells by using the transfection reagent (Santa Cruz). One to two µg of GFP-LC3B (rat) [26] was transfected into 2×10^5^ cells using lipofectamine™2000 according to the supplier's protocol (Invitrogen, CA).

### HDAC Activity

HDAC activity was measured by using a HDAC Colorimetric Assay Kit (MBL international, Woburn, MA).

### Western blotting

All antibodies were from Santa Cruz, except the anti-LC3B and anti-cleaved caspase-3 were obtained from Abgent (San Diego, CA) and Cell Signaling (Beverly, MA), respectively. Western immunoblot analysis was performed using standard methods.

### Chromatin immunoprecipitation (CHIP) assay

The proximal promoter region (−550/−1) of the human *LC3B* gene was analyzed for transcription factor binding sites. Four putative Egr-1 and seven E2F factor binding sites were found (See Figure S1). The primer set 5′-GCTCGGGACAAAAGCAGTT-3′ and 5′-CCCTGAGGTGACGGTTGT-3′ was used to amplify a 427 bp DNA fragment (−550/−124) of the *LC3B* promoter region. After treatment with CSE or TSA, Beas-2B cells were harvested and subjected to the CHIP assay (Active Motif, Carlsbad CA), following the manufacturer's protocol.

### Caspase-3 activity assay

The caspase-3 activity of human lung samples was determined by a Caspase-3 Activity Assay Kit (Calbiochem).

### Statistical analysis

Results were expressed as means±SD from at least three independent experiments. Differences in measured variables between experimental and control group were assessed by using the Student's t test. Statistically significant difference was accepted at *P*<0.05.

## Supporting Information

Figure S1 Supplement to Materials and Methods
(0.01 MB DOC)
