# Supplementary material for: Egr-1 Regulates Autophagy in Cigarette Smoke-Induced Chronic Obstructive Pulmonary Disease
Source: PLoS One. 2008 Oct 2;3(10):e3316. doi: 10.1371/journal.pone.0003316 (PMC2552992; doi:10.1371/journal.pone.0003316)
Supplement: Figure S1 — Supplement to Materials and Methods (0.01 MB DOC) [file pone.0003316.s001.doc]

**Figure S1**

GCTCGGGACAAAAGCAGTTGCGCA**AACGCGCCAA**GGCTGGGCGTCGAGTGAC**CGCGGGCGGA**GGTCACCAGCGGCCA**CTCCCCGGAA**GCCACCCACGGACCACGCGCGCCCCTGCACGCAGAGGGGGCCAGGGCTCCACGGGCGAGCGGCGACCCTGCCTCCCGGAGACGGCGCGGCCTGCCCTGCGCGCCTCAGCCCCGGGTGCCGGCGTCTCGGGCAGCACCACCAAGTCTCTCTGGAGGGGAAAGGATGGTCGGA**TTTGCCCCAT**GTCCCTTCCTCTGACCCCTCCCTCAAGAGTGCCCCGGGACACCCCGCCTGTGGCTCAGCCTCCCCCGCCCCGCGCTGCCATCTCCTCAGGGCCGGGCAGCAGGCTCCCGAGCGCCCACAGACCCGGGGTGCGGCCCAGCCCACAACCGTCACCTCAGGGGCCTCAGGCGCCCAGCGGTGCT**GGGCGGGGC**TGGGGCACGACCGGGAGCATGCGCAGAGCGCGCG**TTTCGCCC**ATCGCGCACGCGCACACACCTGC**TCCGCCCCCAC**GCTGCGTGCCGCTGCT

**Sequence analysis of the LC3B promoter region (-550/-1)**

Putative E2F sites (Bold face) occur at nucleotide positions: -516/-526; -489/-498; -463/-472; -283/-292;

-93/-101; -51/-58; -17/-27.

Putative Egr-1 sites (Underscore) occur at nucleotide positions: -491/-499; -214/-223; -38/-46; -17/-25.

Egr-1 sites at -491/-499; and -17/-25 overlap with putative E2F sites.
